# Extraction, purification, characterization and antioxidant activities of polysaccharides from *Ramaria botrytis* (Pers.) Ricken

**DOI:** 10.1186/s13065-017-0252-x

**Published:** 2017-03-16

**Authors:** Hua Li

**Affiliations:** College of Food Science and Technology, Henan University of Technology, Zhengzhou, 450001 China

**Keywords:** *Ramaria botrytis*, Polysaccharides, Purification, Antioxidant activities

## Abstract

**Background:**

*Ramaria botrytis* (Pers.) Ricken, a member of the family Clavariaceae, has been widely prescribed for anti-aging and improving immunity. To extract and purify the polysaccharides, the main constituent of the fruiting-body, from *R. botrytis* and explore antioxidant activities was great significant.

**Results:**

*Ramaria botrytis* polysaccharides (RBP) was extracted with water at 88.47 °C for 1.42 h with a solution to sample ratio of 10.94 mL g^−1^ employing response surface methodology. Four purified fractions, RBP-1, RBP-2, RBP-3, and RBP-4, were obtained from column chromatography of DEAE-52 and Sephadex G-100. Among these four purified fractions, RBP-1, RBP-2, RBP-4 were mainly composed of glucose, while RBP-3 contained 41.36% mannose and 28.96% glucose. The molecular weights of RBP-1, RBP-2, RBP-3 and RBP-4 were 6.48, 36.12, 96.72 and 8.34 kDa, respectively. These four fractions are also tested for antioxidant activities in vitro, RBP-4 exhibited strong assay of reducing power and high scavenging activity on DPPH radical, while RBP-3 showed the stronger ability of hydroxyl radical scavenging activity.

**Conclusions:**

Response surface methodology was successfully applied to optimize the ultrasonic extraction of polysaccharides from *R. botrytis*. RBP is an efficient natural antioxidant.

## Background

Edible mushrooms commonly used as food, flavoring substances or folk traditional medicines, are well-known for their abundant nutrients: carbohydrates, proteins, vitamins, minerals, characteristic flavour components, and other bioactive components [1]. Meanwhile, Products from wild and cultivated edible mushrooms, have acquired considerable attention toward their biological functions, such as improving immunity, antioxidant, anti-cancer and anti-viral activities due to their functional constituents [2–4].

Extensive studies have been done with the structure and bioactivity mechanism of natural polysaccharides and their conjugates, which have been used in food and medicine for a long time [5, 6]. Numerous researches demonstrated that plenty of natural polysaccharides were good at protecting human bodies from oxidative damage in the growth and development of living organism [7–9]. Therefore, natural polysaccharides are considered as a potential resource of novel antioxidants, and the mechanism of polysaccharide are in need of further research [6, 10].

*Ramaria botrytis* (Pers.) Ricken, one of mushrooms widely consumed as edible food especially prevailing Asian countries including China, mainly due to its special favor and rich nutrients. It is known as cauliflower coral and belongs to Clavariaceae [11]. Polysaccharide, water soluble and water insoluble, is one of the most important bioactive substances in *R. botrytis*. Recent research revealed that two water insoluble glucans had been isolated from the alkali extract of the fruit bodies of *R. botrytis* [11]. In this paper, the extraction, purification, characterization and antioxidant activities of polysaccharides isolated from *R. botrytis* is described. This study aims to purify fractions of water soluble polysaccharides, analyze their preliminary characteristics and investigate their antioxidant activities.

## Experimental procedures

### Materials and chemicals

The samples of *R. botrytis*, collected by the author in Ailao mountains, Yunnan Province, China, in August 2013. Identification of the mushrooms was performed by Prof. Li Yu, the academician of Jilin Agricultural University. Removed impurities and cleaned with water, the samples were air-dried to constant weight at 60 °C. Then the dried sample was ground into fine powder and screened through a 40 mesh sieve. The powder was prepared for the subsequent studies.

Analytical grade of 2, 2-diphenyl-1-picryl-hydrazyl (DPPH) and 1, 10-phenanthroline was purchased from the Sigma-Aldrich Trading Limited Corporation (Shanghai, China) and the Kermel Chemical Corporation (Tianjin, China), respectively. Other reagents used in this study were of analytical grade.

### Box–Behnken factorial design (BBD) for the extraction of RBP

Box–Behnken factorial design was used as interaction design to explore the effect of the main independent variables. Based on the preliminary single factor experiment and BBD principle, a three-factor-three-level BBD was employed in this study. Three extraction variables: X_1_ (water to raw material ratio), X_2_ (extraction temperature), and X_3_ (extraction time) (Table 1) were viewed as the independent variables, and the purity of the RBP was the dependent variable in this design.

**Table 1:** Independent variables and their levels for the extraction of RBP

Independent variables	Factor		
	−1	0	1
Water to raw material ratio (mL/g)	10	15	20
Extraction temperature (°C)	70	80	90
Extraction time (h)	1.0	1.5	2.0

The result of the BBD contained 17 experimental points, including twelve factorial points and five axial points. The five axial points were for pure error estimation in the test. The non-linear quadratic model produced in the response surface by Design Expert 8.0 is shown in Eq. (1) [12]:

1 $$y = \beta_{{k_{0} }} + \mathop \sum \limits_{i = 1}^{3} \beta_{{k_{i} }} + \mathop \sum \limits_{i = 1}^{3} \beta_{{k_{ii} }} X_{i}^{2} + \mathop \sum \limits_{i < j = 2}^{3} \beta_{{k_{ij} }} X_{i} X_{j}$$

where y is the dependent variable, $\beta_{{k_{0} }}$ is the constant, $\beta_{{k_{i} }}$, $\beta_{{k_{ii} }}$, and $\beta_{{k_{ij} }}$ represent the linear regression coefficients of variables, quadratic and interaction terms, respectively; X_i_ and X_j_ are the independent variables wherein i and j are the levels of the independent variables (i ≠ j). The regression analysis and analysis of variances (ANOVA) helped predict the polynomial model to investigate complex processes. The fitted polynomial equation, aiming at visualizing the relationship between the response and experimental levels of each factor, developed the final response surfaces and deduced the optimum conditions [13, 14]. The regression coefficients from the regression model generated different dimensional and contour maps. The predicted values, calculated by Statistica (Version8.0, USA), aimed at estimating the statistical significance of the independent variables. The polysaccharide content of crude RBPs was determined by phenol–sulfuric acid method [15].

### Analytical method validation

The total content of polysaccharide in *R. botrytis* was analyzed by phenol–sulfuric acid method using glucose as standard [15]. The regression equation was *Y* = 0.0124*x* *−* 0.0032 with the correlation coefficient as 0.9926, where *Y* represents absorbance, *x* represents the concentration of glucose or RBP. A linear relationship between the absorbance and the polysaccharide quantity was observed within the range of 0–40 μg mL^−1^, detected at 490 nm wavelength.

The extraction method was validated in terms of precision and accuracy. The precision was estimated by analyzing the intra-day (repeatability) and inter-day (intermediate) precision variations. The repeatability was evaluated by testing standard solution at three different concentrations (0.05, 0.10 and 0.20 mg mL^−1^) with five replicates during one day, and the intermediate precision was evaluated by testing standard solution at three different concentrations (0.05, 0.10 and 0.20 mg mL^−1^) for three days. The accuracy was evaluated with the spiked recovery test. Three different standards (0.05, 0.10 and 0.20 mg mL^−1^) were added to blank sample separately for further extraction and analysis.

### Preparation of crude RBP

The Sevage solution was adopted to remove the proteins in the crude RBP after extracted under the optimal condition. The deproteinized RBP was extracted with the reaction mixture (chloroform: butyl alcohol, 5:1) for three times. After centrifugation (15 min, 4000 rpm, 20 °C), ethanol was added into the supernatant until the final concentration of ethanol was 50%. The mixture was standing at 4 °C for 18 h, then centrifugal separated at 4000 rpm for 15 min. The supernatant was collected and repeated the same procedure until the final concentration of ethanol was 60, 75, 85 and 95%. The precipitate was collected, freeze-dried and accurately weighed respectively, for further study.

### Purification of RBPs

Crude RBP was purified sequentially by DEAE-52 cellulose and Sephadex G-100 filtration chromatography according to a previous study with little modifications [16]. In detail, the RBP solution (3 mL, 10 mg mL^−1^) was applied tardily to a column (2.6 × 40 cm) of DEAE-52 cellulose. The column was stepwise eluted with 0, 0.1, 0.3 and 0.5 mol L^−1^ NaCl solutions at a flow rate of 1.0 mL min^−1^. Then the obtained elutes (5 mL per tube) were collected by the automatic collector. According to the phenol–sulfuric acid method, each fraction of polysaccharides of RBP was collected. Repeat the process and collect the same fractions together. Each fraction was concentrated, dialyzed and freeze-dried. The solution (2 mL, 30 mg mL^−1^) of each fraction was further purified through the Sephadex G-100 column (2.6 × 60 cm). The elutes were collected automatically eluted with deionized water, then concentrated and freeze-dried for further research.

### Characterization of RBP

The monosaccharide composition of RBP-1, RBP-2, RBP-3 and RBP-4 were analyzed by high performance anion exchange chromatography (Dionex ICS-3000, Sunnyvale, CA, USA) in combination with a carbopac PA-1 ion exchange column (4 × 250 mm).

The average molecular weights of polysaccharide fractions were determined by gel permeation chromatography (GPC). Each sample (2.0 mg) was dissolved in distilled water (2 mL), passed through a 0.45 μm filter, and then applied to a column of gel-permeation chromatographic at a flow rate of 0.5 mL min^−1^ [14]. The calibration curve was conducted by reference of the dextrans with various molecular weight (P-400, P-100, P-50, P-10, and P-5).

### Determination of antioxidant activities

#### DPPH radical-scavenging activity

The DPPH radical-scavenging activity of RBPs was assayed based on a reported method [14] with little modification. A series of sample solutions (0.2, 0.4, 0.6, 0.8, 1.0 and 1.2 mg mL^−1^) were prepared by dissolving polysaccharide samples into distilled water. DPPH powder was dissolved in ethanol (0.1 mM). Aliquots of 1 mL of the sample solution and 1 mL of DPPH solution were mixed until homogeneity in a cuvette and incubated 20 min in the dark. Then the absorption was measured at 517 nm to detect the reduction of DPPH in the cuvette. Ascorbic acid was used as a positive standard. The DPPH radical scavenging activity of RBPs was expressed by Eq. (2):

2 $$DPPH\,radical\,scavenging\,activity\,(\% ) = \left( {1 - \frac{{A_{1} - A_{3} }}{{A_{2} }}} \right){ \times }100$$

where* A*_1_ is the absorbance of the reaction solution which contains 1 mL of sample and 1 mL of DPPH solution,* A*_3_ is the absorbance of the solution including 1 mL of sample and 1 mL of ethanol, and* A*_2_ is the absorbance of the solution including 1 mL of DPPH and 1 mL of ethanol.

#### Hydroxyl radical-scavenging activity

The assay of hydroxyl radical-scavenging activity of RBPs was carried out according to a reported method described previously [17]. Briefly, 1 mL of distilled water, 1 mL of 1,10-phenanthroline (0.75 mM), 1 mL of Fe_2_SO_4_ (0.75 mM) and 1 mL of H_2_O_2_ (0.01%) were dissolved into 2 mL of phosphate buffer (pH 7.4) and mixed thoroughly. Incubated at 37 °C for 60 min, the mixture solution was used as the blank solution. The control solution was prepared under the similar sequence, only 1 mL of distilled water instead of 1 mL of H_2_O_2_. The four fractions of polysaccharides were dissolved in distilled water, yielding a series of sample concentrations (0.2, 0.4, 0.6, 0.8, 1.0 and 1.2 mg mL^−1^), respectively. According to the same procedure, the sample solution was prepared, wherein 1 mL of distilled water was replaced by 1 mL of polysaccharide solution. Then, the absorbance of the blank (B_blank_), control (B_control_), and sample solutions (B_sample_) was determined at 510 nm. The results were calculated by Eq. (3):

3 $$Hydroxyl \, radical \, scavenging \, activity \, \left( \% \right) = \frac{{B_{sample} { - {\rm B}}_{blank} }}{{B_{control} { - {\rm B}}_{blank} }}{ \times }100$$

#### Reducing power

The reducing power was determined by the method [18] with some modifications. The four RBPs were dissolved in distilled water to form various sample solutions (0.5, 1.0, 1.5, 2.0, 2.5 and 3.0 mg mL^−1^). A volume of 2 mL sample solution was added into 2.5 mL phosphate buffer (0.2 M, pH 6.6) and 2.5 mL of potassium ferricyanide (1%, w/v). Incubated at 50 °C for 20 min, 2.5 mL of trichloroacetic acid (TCA) was added to the mixture and centrifuged at 3000 rpm for 10 min. The final mixture solution was formed by adding 2.5 mL distilled water and 0.5 mL ferric chloride (0.1%, w/v) to 2.5 mL of the supernatant. The absorbance of the reaction mixture was measured at 700 nm. Ascorbic acid was used as the positive control. A higher absorbance indicates a stronger reducing power of the sample.

## Results and discussion

### Optimization for the extraction parameters of RBP

#### Model fitting preliminary

Relying on the 17 experimental points designed by the BBD (Design Expert 8.0, USA), the corresponding yield of RBP were obtained according to the preliminary standard curve. The yield of RBP ranged from 5.97 to 9.90% (Table 2). The correlation between response variables and test variables was expressed by the following second-order polynomial equation [19]:

$$Y = \, 8.81 \, + \, 0.27X_{1} + \, 1.61X_{2} + \, 0.21X_{3} + \, 0.075X_{1} X_{2} + \, 0.12X_{1} X_{3} + \, 0.15X_{2} X_{3} - 0.81X_{1}^{2} - 0.13X_{2}^{2} - 0.78X_{3}^{2}$$

**Table 2:** The Box–Behnken design and the yield of *Ramaria botrytis* polysaccharide

Run	*X*_1_/water to raw material ratio (mL g^−1^)	*X*_2_/extraction temperature (°C)	*X*_3_/extraction time (h)	Extraction yield (%)	Predicted yield (%)
1	10 (−1)	90 (1)	1.5 (0)	9.10	9.11
2	20 (1)	70 (−1)	1.5	6.47	6.46
3	20	80 (0)	2.0 (1)	7.97	7.82
4	15 (0)	80	1.5	8.57	8.81
5	15	80	1.5	8.45	8.81
6	15	70	1.0 (−1)	6.50	6.25
7	20	90	1.5	9.90	9.80
8	15	80	1.5	8.87	8.81
9	15	70	2.0	6.20	6.36
10	15	80	1.5	9.07	8.81
11	15	90	2.0	9.60	9.85
12	10	80	1.0	6.70	6.86
13	10	80	2.0	7.28	7.02
14	10	70	1.5	5.97	6.07
15	15	90	1.0	9.30	9.14
16	20	80	1.0	6.90	7.16
17	15	80	1.5	9.07	8.81

where *Y* represents the yield of RBP (%), *X*_1_, *X*_2_ and *X*_3_ represent ratio of water to solid, extraction temperature and extraction time, respectively.

The results of the analysis of variance (ANOVA) for the quadratic regression model were shown in Table 3. The purity coefficients (R^2^) of the determination was 0.9749, which indicated that only 1.30% of the total variance was not explained by the model. At the same time, the adjusted determination coefficient (adj-R^2^ = 0.9626), which was very close to R^2^, which demonstrated the model was extremely significant. This result showed high consistency between the experimental values and theoretical values predicted by the polynomial regression model. The *p* values were able to confirm the significance of each coefficient, which in turn may indicated interaction patterns among the variables [14]. The corresponding coefficient was more significant if the *p* value was smaller. Accordingly, the model was extremely significant (*p* < 0.05). Meanwhile, *X*_1_, *X*_3_, *X*_1_^2^, *X*_2_^2^ were significantly different (*p* < 0.05), while *X*_2_, *X*_3_^2^, *X*_1_ *X*_2_, *X*_1_ *X*_3_ and *X*_2_ *X*_3_ were not significantly different (*p* > 0.05). The parameter, lack of fit, was used to express the difference between the model and the experiment. It was beneficial to the model without any significance in the lack of fit.

**Table 3:** ANOVA for the quadratic regression model in BBD

Source	Sum of squares	DF	Mean squares	*F* value	*p* value
Model	27.31	9	3.03	30.20	<0.0001
*X*_1_	0.60	1	0.60	5.97	0.0446
*X*_2_	20.35	1	20.35	202.53	<0.0001
*X*_3_	0.34	1	0.34	3.39	0.1083
*X*_1_ *X*_2_	0.022	1	0.022	0.22	0.6505
*X*_1_ *X*_3_	0.06	1	0.06	0.60	0.4649
*X*_2_ *X*_3_	0.09	1	0.09	0.90	0.3755
*X*_1_^2^	2.81	1	2.81	27.95	0.0011
*X*_2_^2^	0.07	1	0.07	0.70	0.4304
*X*_3_^2^	2.54	1	2.54	25.28	0.0015
Residual	0.70	7	0.10		
Lack of fit	0.38	3	0.13	1.54	0.3336
Pure error	0.33	4	0.081		
Cor total	28.01	16			
R^2^	0.9749		Adj-R^2^	0.9626	

#### Optimization for the extraction of RBP

**Fig. 1:**
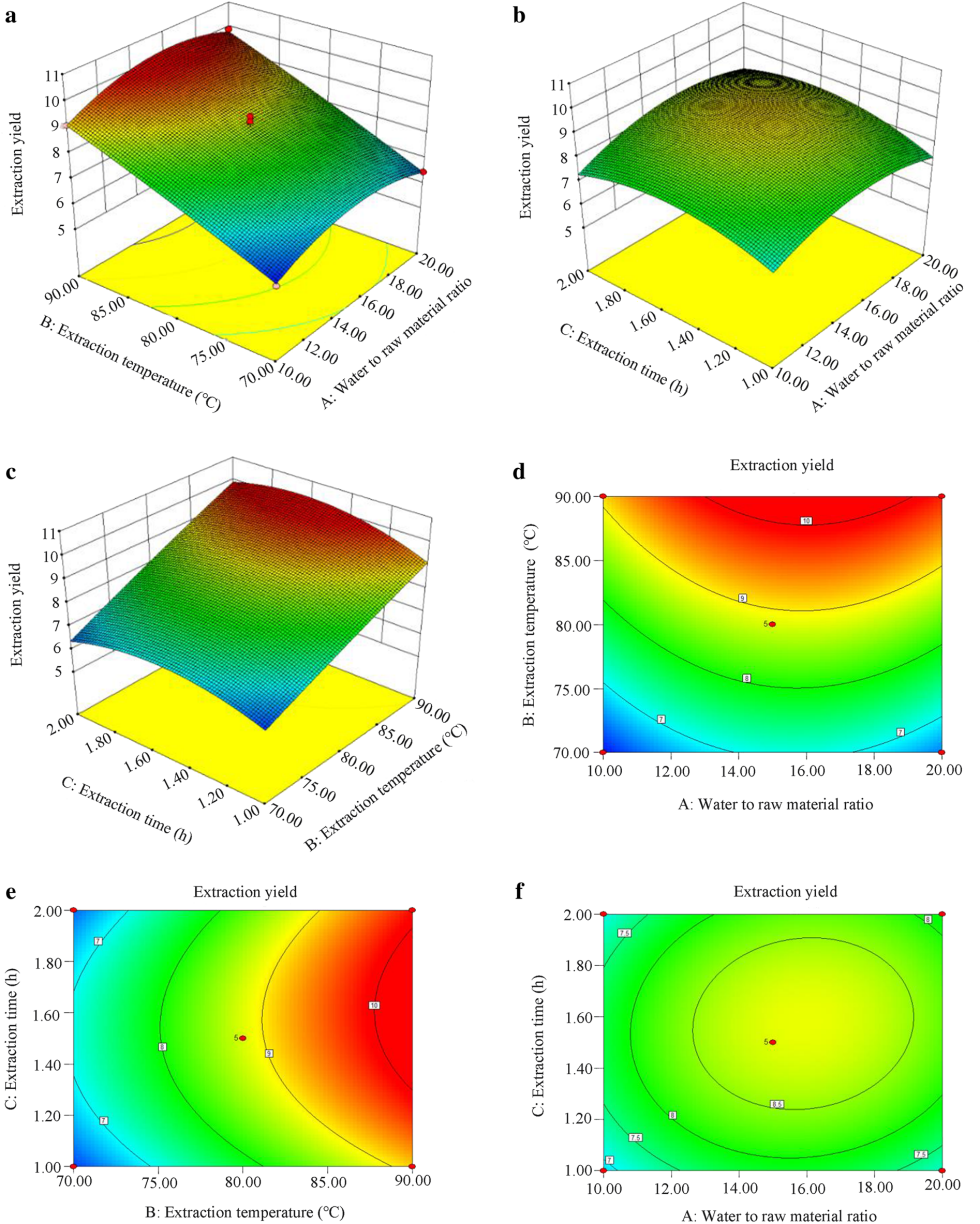
Three-dimensional plots (**a**, **b**, **c**) and their corresponding contour plots (**d**, **e**, **f**) showing the effect of each two independent variables on the yield of RBP

Generated by Design-Expert, these three-dimensional plots and their corresponding contour plots (Fig. 1), which were graphical representations of the quadratic regression equation, presented the interactions of three variables (Table 1) better. By keeping another variable at its zero level, these types of contour plots visualized whether the interactions between the two variables were significant or not. According to that method, these 3D response surfaces and 2D contour plots provided the significance degree between each two variables. Correspondingly, they facilitated the generation of the optimum experimental combination. The optimum experimental variables for the extraction of RBP were as follows: extraction temperature 88.47 °C, extraction time 1.42 h and ratio of water to solid 10.94 mL g^−1^. Among the three effective parameters, the extraction time was the most significant factor during the extraction of RBP. Between the other parameters, the ratio of water to solid was more significant than the extraction temperature.

#### Verification of the model

The relative standard deviation (RSD) value of repeatability was 3.25%, and the RSD value of intermediate precision was 2.68%, which showed the precision of instruments was good. The spiked recoveries of glucose were 91.20–104.30%. In summary, the method was effective and reliable. The polysaccharide yield was 9.08% according to the optimal extraction condition, in which the extraction temperature 90 °C, extraction time 1.5 h and ratio of water to solid 11.00 mL g^−1^.

### Fractional precipitation of polysaccharides

The yield of the precipitation was 58.06, 12.08, 18.78 and 11.08%, as the concentration of ethanol 50, 75, 85 and 95%. No precipitate appeared when the concentration of ethanol was up to 95%. From the yield, the polysaccharide collected with the concentration of ethanol 50% was the main component and was acted as crude polysaccharide to purify further.

### Purification of crude RBP

**Fig. 2:**
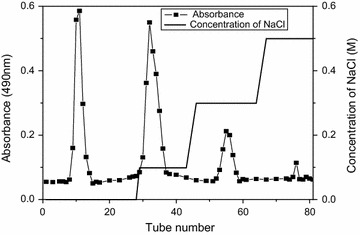
0, 0.1, 0.3, 0.5 M NaCl stepwise elution curve of crude RBP by DEAE-52 column

**Fig. 3:**
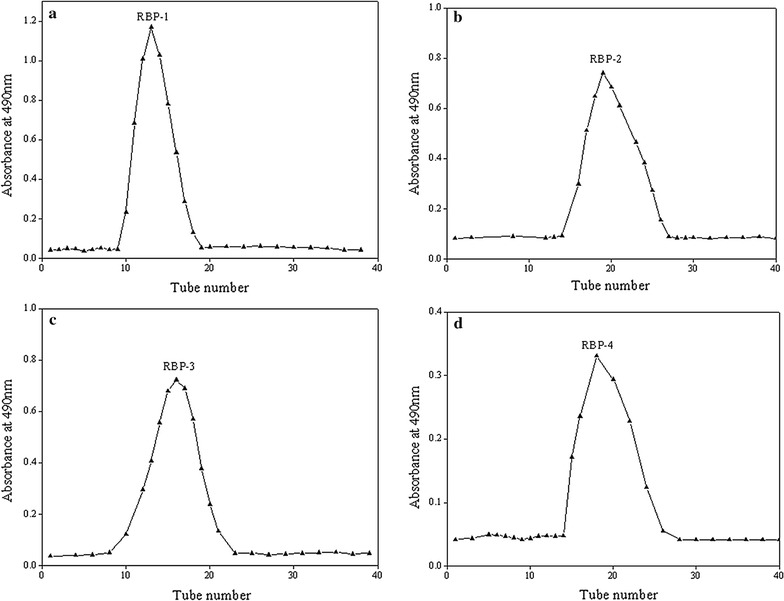
Distilled water elution curve of each fraction **a** RBP-1, **b** RBP-2, **c** RBP-3, **d** RBP-4 on Sephadex G-100 column

Crude polysaccharide of 20 g was purified firstly by a DEAE-52 cellulose column, which could isolate polysaccharides with negative charges from the crude polysaccharide. After the elution with 0, 0.1, 0.3 and 0.5 mol L^−1^ NaCl solutions, four independent peaks in Fig. 2 appeared using the phenol–sulfuric acid method. Each fraction was collected, concentrated, dialyzed, freeze-dried and loaded to a column of Sephadex G-100, which was eluted with deionized water. Finally, each fraction produced a single elution peak (Fig. 3a–d), which defined as RBP-1, RBP-2, RBP-3 and RBP-4, respectively.

### Characterization of RBP

#### Monosaccharide composition of RBP

The monosaccharide composition of RBP-1, RBP-2, RBP-3 and RBP-4 was analyzed by high performance anion exchange chromatography and a carbopac PA-1 ion exchange column. From results shown in Table 4, different purified fractions had different monosaccharide compositions. RBP-1 contained only two kinds of monosaccharides: gluctose (88.24%) and galactose (11.76%). RBP-2 was mainly composed of glucose. Meanwhile, the contents of galactose, mannose and xylose in RBP-2 were much lower than those in RBP-1 and RBP-4. Little arabinose only existed in RBP-3.

**Table 4:** Monosaccharide composition for RBP-1, RBP-2, RBP-3, RBP-4

Samples	RBP-1	RBP-2	RBP-3	RBP-4
Weight (g)	4.42	8.87	1.56	0.35
Glucose	88.24%	95.42%	28.96%	65.62%
Galactose	11.76%	1.94%	14.37%	15.15%
Mannose	–	1.98%	41.36%	–
Arabinose	–	–	0.31%	15.28%
Xylose	–	0.66%	15.01%	3.95%

–, not detected

#### Molecular weight determination of RBPs

The molecular weight of RBP-1, RBP-2, RBP-3, and RBP-4 was determined by GPC method. According to the different molecular weight of dextran standards, the average molecular weights of RBP-1, RBP-2, RBP-3 and RBP-4 were 6.48, 36.12, 96.72 and 8.34 kDa, respectively.

### Antioxidant activity in vitro of RBP

#### Scavenging activity on DPPH radical of RBP

**Fig. 4:**
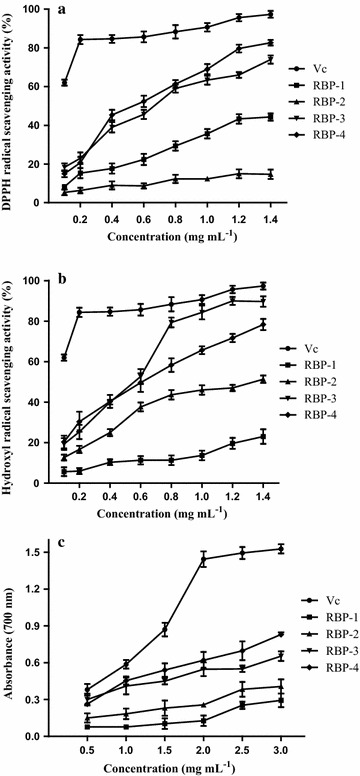
Scavenging activities on **a** DPPH radical, **b** hydroxyl radical, **c** reducing power assay for RBP-1, RBP-2, RBP-3, RBP-4 at various concentrations. Data shown were mean ± standard deviation (n = 3)

Acted as hydrogen donors, DPPH, which owns a proton free radical with a characteristic absorption, has been widely used to evaluate antioxidant activity of polysaccharides [4, 8]. The scavenging ability of four polysaccharides for DPPH∙ radical is shown in Fig. 4a and ascorbic acid was the positive control. The results indicated that RBP-4, RBP-3 and RBP-3 displayed concentration dependent radical scavenging effects although weaker than that of Vc in the same concentration, and the order was RBP-4 > RBP-3 > RBP-1 > RBP-2. Along with the increased concentration of each polysaccharide, the DPPH∙ scavenging ability increased. At 1.4 mg mL^−1^ of RBP-4, the DPPH scavenging percentage was 82.67%, and less than the ascorbic acid control 15%, while the scavenging percentage of RBP3, RBP1and RBP-2 was 74.01, 44.33 and 14.67%. RBP-2 showed lowest effect on DPPH, perhaps due to its special structure, that should be studied further.

#### Assay of hydroxyl radical scavenging activity

The hydroxyl radical, which has high reactivity and a very short half-life of approximately 10^−9^ s in vivo, is the most reactive and dangerous compound generated through the Fenton reaction to organisms [8]. The hydroxyl radical-scavenging activity of RBP-1, RBP-2, RBP-3, RBP-4 and ascorbic acid determined at 510 nm were depicted in Fig. 4b. The results showed the scavenging activity of RBP-3 was higher than RBP-4, RBP-2, RBP-1, but lower than ascorbic acid. The hydroxyl radical-scavenging activity of ascorbic acid and all the polysaccharides increased gradually as their concentrations increased. With the increase of amount in the range of 0–1.2 mg mL^−1^, hydroxyl radical-scavenging activityof each compound increased, whereas the activity of RBP-3 (90%) was approximatelythe same as ascorbic acid (95.33%) at the concentration of 1.2 mg mL^−1^.

#### Assay of reducing power

Served as a significant indicator of its potential antioxidant activity, the reducing power of a compound may directly reflect the production condition of electron donor [20, 21]. The reducing power of RBP-1, RBP-2, RBP-3, RBP-4 and ascorbic acid determined at 700 nm is depicted in Fig. 4c. Ascorbic acid is a well-recognized reducing agent. As shown in the figure, the reducing power of ascorbic acid increased quickly as the concentration increased from 0.2 to 1.2 mg mL^−1^. All four samples showed higher reducing power with the increasing of their concentrations, but much lower than ascorbic acid. RBP-4 had the strongest reducing power among the four fractions.

## Conclusion

It can be concluded that the water-soluble and purified polysaccharides from the sporocarp of *R. botrytis* could be obtained with the optimized method. Firstly, The BBD method provided the optimal extraction condition of the crude polysaccharide. And the crude polysaccharide was eluted and purified by two column chromatographies of DEAE-52 and Sephadex G-100 successively. Four purified fractions of polysaccharides, RBP-1, RBP-2, RBP-3 and RBP-4 were obtained in this study, which average molecular weights were 6.48, 36.12, 96.72 and 8.34 kDa, respectively. Moreover, RBP-1, RBP-2, RBP-4 were mainly composed of glucose, with a percentage of 88.24, 95.42 and 65.62%, respectively; while RBP-3 contained 41.36% mannose, 28.96% glucose, 15.01% xylose and 14.37% galactose. Furthermore, the antioxidant activity tests showed that RBP-4 had strong assay of reducing power and high scavenging activity on DPPH radical, while RBP-3 exhibited the strongest ability of hydroxyl radical scavenging activity. All the results implied that RBP could be a promising new natural antioxidant in food industry or drug therapies.
